# The effect of metformin on survival of patients with pancreatic cancer: a meta-analysis

**DOI:** 10.1038/s41598-017-06207-x

**Published:** 2017-07-19

**Authors:** Xiaogang Li, Tong Li, Zhiqiang Liu, Shanmiao Gou, Chunyou Wang

**Affiliations:** 1 Department of Pancreatic Surgery, Union Hospital, Tongji Medical College, Huazhong University of Science and Technology, Wuhan 430022, China; 2 Department of Hepatobiliary Surgery, Union Hospital, Tongji Medical College, Huazhong University of Science and Technology, Wuhan 430022, China; 3 Department of General Surgery, Xiangyang Central Hospital, Hubei University of Arts and Science, Xiangyang 441021, China

## Abstract

We conducted a meta-analysis to analyse the effect of metformin on survival of pancreatic cancer patients at various stages. We performed a systematic search of PubMed, Embase, Cochrane, and Web of Science to identify all relevant studies. Summary hazard ratios (HR) of survival and 95% confidence intervals (95% CI) were calculated with a fixed or random effects model according to inter-study heterogeneity. Nine retrospective cohort studies and two randomized controlled trials (RCTs) were eligible. There was a significant improvement in survival (HR = 0.86, 95% CI 0.76–0.97; *P* < 0.05) in the metformin group compared with control. Subgroup analysis indicated that metformin improved survival in patients with resection (HR = 0.79, 95% CI 0.69–0.91; *P* < 0.05) and patients with locally advanced tumors (HR = 0.68, 95% CI 0.55–0.84; *P* < 0.05) but not in patients with metastatic tumors, even when RCT data were included (HR = 0.99, 95% CI 0.70–1.40; *P* > 0.05), or were excluded (HR = 0.89, 95% CI 0.61–1.31; *P* > 0.05). This meta-analysis indicated that the effect of metformin does correlate with tumor stage but should be prudently considered given the limited and variable studies performed to data.

## Introduction

Pancreatic cancer is among the most lethal of malignancies^1–4^. Each year, 53,000 new cases are diagnosed with this disease, which leads to approximately 42,000 deaths annually in the United States^5^. Limited advances have been made regarding treatment developments and the disease prognosis remains poor, with the 5-year overall survival rate ranging from 3–6% with a median survival of < six months^6–9^. Even in patients at early stages of the disease who receive surgical resection, the 5-year survival rate is no more than 24% due to a high rate of local recurrence and metastasis^10–13^.

Type 2 diabetes mellitus is considered to play important roles in tumorigenesis and development of pancreatic cancer. Insulin resistance and up-regulated insulin-like growth factor I in type 2 diabetes mellitus are proposed to be the mechanisms underpinning the disease onset. Metformin is an antidiabetic drug commonly prescribed for patients with type 2 diabetes mellitus. Recently, increasing evidence has indicated that metformin can decrease the risk of developing pancreatic cancer in patients with concurrent type 2 diabetes mellitus^14, 15^. However, this effect has not been observed with other antidiabetic agents. Moreover, subsequent experimental studies have also confirmed the antitumor effect of metformin both *in vitro* and *in vivo*, which provided a rationale for clinical use of metformin in cancer treatment. However, there is still no consensus relating to the use of metformin in pancreatic cancer.

Several meta-analyses have shown that metformin decreases the risk of pancreatic cancer^16–21^ but only one meta-analysis has been conducted, which explored the association between metformin and survival of pancreatic cancer patients^22^. In the meta-analysis, four studies were included and the results showed no evidence for metformin being either harmful or beneficial with regard to survival of pancreatic cancer patients (HR = 0.80, 95% CI 0.62–1.03; *P* > 0.05). However, some studies have suggested that the effect of metformin delivery varied among pancreatic cancer patients with different tumor stage, and therefore analysis of subgroups by tumor stage was performed^23–27^. In addition, all studies involved in the meta-analysis were cohort studies, and the results of two RCTs^28, 29^ have been published recently. Consequently, further meta-analysis is required. To this end, we performed a meta-analysis on nine cohort studies^23–27, 30–33^ and two RCTs in order to determine the effect of metformin on survival of pancreatic cancer patients. In addition, subgroup analysis was performed to determine whether tumor stage affected the response to metformin.

## Results

### Literature search

**Figure 1:**
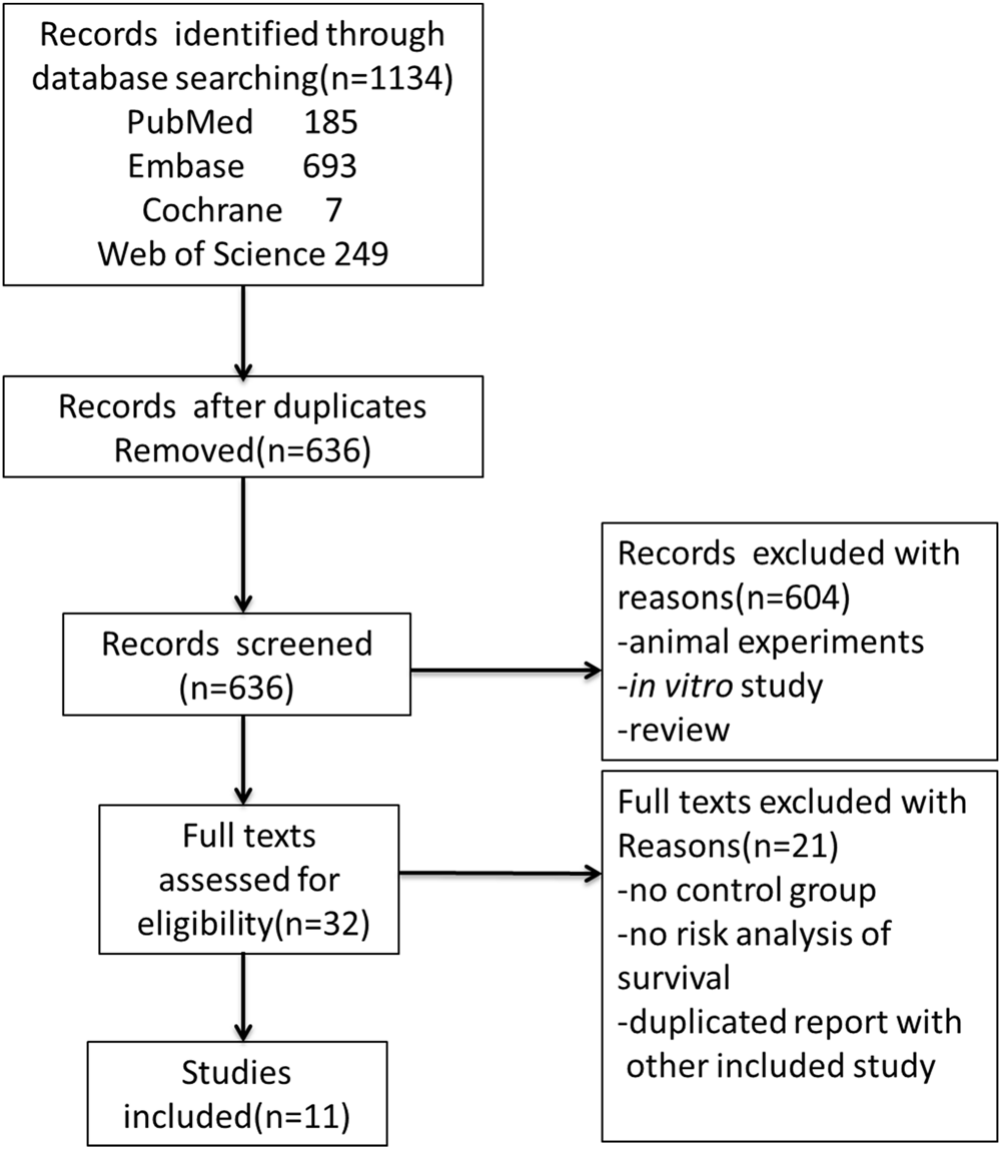
PRISMA flowchart summarizing the study identification and selection.

The selection process for the literature search is summarized in Fig. 1. Initially, a total of 1134 articles were identified form the search of the four databases. After reviewing titles and abstracts for relevance, 32 articles were evaluated further for eligibility. Of the 32 articles, 21 were excluded: nine articles were conference abstracts without detailed data; five articles pertained to the same study; three articles did not report the outcomes of HR, and only provided the mean survival time; and in another four articles, there were no control groups. Thus, nine cohort studies and two RCTs were included in this systematic review. The blinding method, randomization, generation of random sequences, and allocation concealment of the two RCTs are shown in Supplementary Table S1 and Figure S1.

### Quality of studies

The Jadad score^34^ of the two RCTs was 5 and 2, respectively. The trial of Reni *et al*. was of low quality (≤2), and the trial of Kordes *et al*. was of high quality (>2), according to the Jadad score. The risks of bias in nine retrospective cohort studies are shown in Supplementary Table S2. Of the nine retrospective cohort studies, the NOS scores of five studies were seven, and the remaining four studies were eight. All of the retrospective cohort studies were of high quality (≥7).

### Study characteristics

The characteristics of 11 included studies are shown in Table 1. In the two RCTs included in this meta-analysis, a total of 181 patients with locally advanced or metastatic pancreatic cancer that could not be treated with resection, were studied. Patients in the study group of Reni *et al*. were treated with cisplatin, epirubicin, capecitabine, gemcitabine (PEXG) and metformin. In the study of Kordes *et al*., patients in the study group were treated with gemcitabine, erlotinib, and metformin given orally as a step-up dose. Meanwhile, patients in the control group received gemcitabine, erlotinib, and placebo.

**Table 1:** Characteristics of included studies.

study	design	tumor stage	size	treatment or metformin exposure in study group	intervention in control group	HR(95% CI)
Ambe 2015	RCS	resectable	44	ongoing metformin use	never used metformin	0.54(0.16–1.86)
Amin 2016	RCS	all stages	1916	use in the 6 months prediagnosis.	diabetic medications other than metformin	0.88(0.81–0.96)
Cerullo 2016	RCS	resectable	3396	use following surgery	no use after surgery	0.79(0.67–0.93)
Chaiteerakij 2016	RCS	all stages	980	ever use	ever use	0.93(0.81–1.07)
Choi 2016	RCS	LA; metastatic	349	unclear	unclear	0.70(0.49–1.99)
Hwang 2013	RCS	LA; metastatic	516	use at any time in the peridiagnosis period	no use during peridiagnosis period	1.11(0.89–1.38)
Kordes 2015	RCT	LA; metastatic	121	GE + metformin	GE + placebo	1.05(0.72–1.55)
Kozak 2016	RCS	resectable	171	continuous use from first consult to discharge postsurgery	never use or discontinued use	0.60(0.21–1.67)
Lee 2016	RCS	all stages	237	cumulative use ≥ 1 month post-diagnosis	use < 1 month or never use	0.61(0.46–0.81)
Reni 2016	RCT	metastatic	60	PEXG + metformin	PEXG	1.56(0.87–2.80)
Sadeghi 2012	RCS	all stages	302	ever use	never use	0.64(0.48–0.86)

Abbreviations: RCS = retrospective cohort study; PA = pancreatic adenocarcinoma; peridiagnosis period = between 6 months prediagnosis and one month postdiagnosis; GE = gemcitabine + erlotinib; PEXG = cisplatin + epirubicin + capecitabine + gemcitabine; all stages = resectable + locally advanced + metastatic; LA = locally advanced.

In the nine retrospective cohort studies, one trial did not offer clear definition of metformin exposure, and the other eight trials gave different definitions for metformin exposure. Two trials defined metformin exposure as ever having used it, while one trial defined exposure as ongoing metformin use. In addition, one trial defined metformin exposure as use in the six months before diagnosis, while another trail defined exposure as ever having used the drug in the peri-diagnosis period. Moreover, one trial did not define metformin exposure clearly, yet the entire cohort of patients in the study group was recorded as having been treated with the drug following surgery. The study of Kozak *et al*. defined metformin exposure as continuous use from the first consultation to discharge after surgery, and the study by Lee *et al*. defined exposure as cumulative use at one month or longer after diagnosis.

The studies were conducted in four countries: the USA, Italy, Korea, and the Netherlands. In addition, most of these were performed in the last two years. In total, 8089 participants were included in these studies. Among the 8089 participants, 2792 were included in the metformin use group, while 5267 cases were included in the non-metformin use group. Three studies included patients at the resectable stage; one RCT included patients at the metastatic stage; another three studies included patients at a locally advanced or metastatic stage; and four studies included patients at either resectable, locally advanced, or metastatic stage.

### Meta-analysis

**Figure 2:**
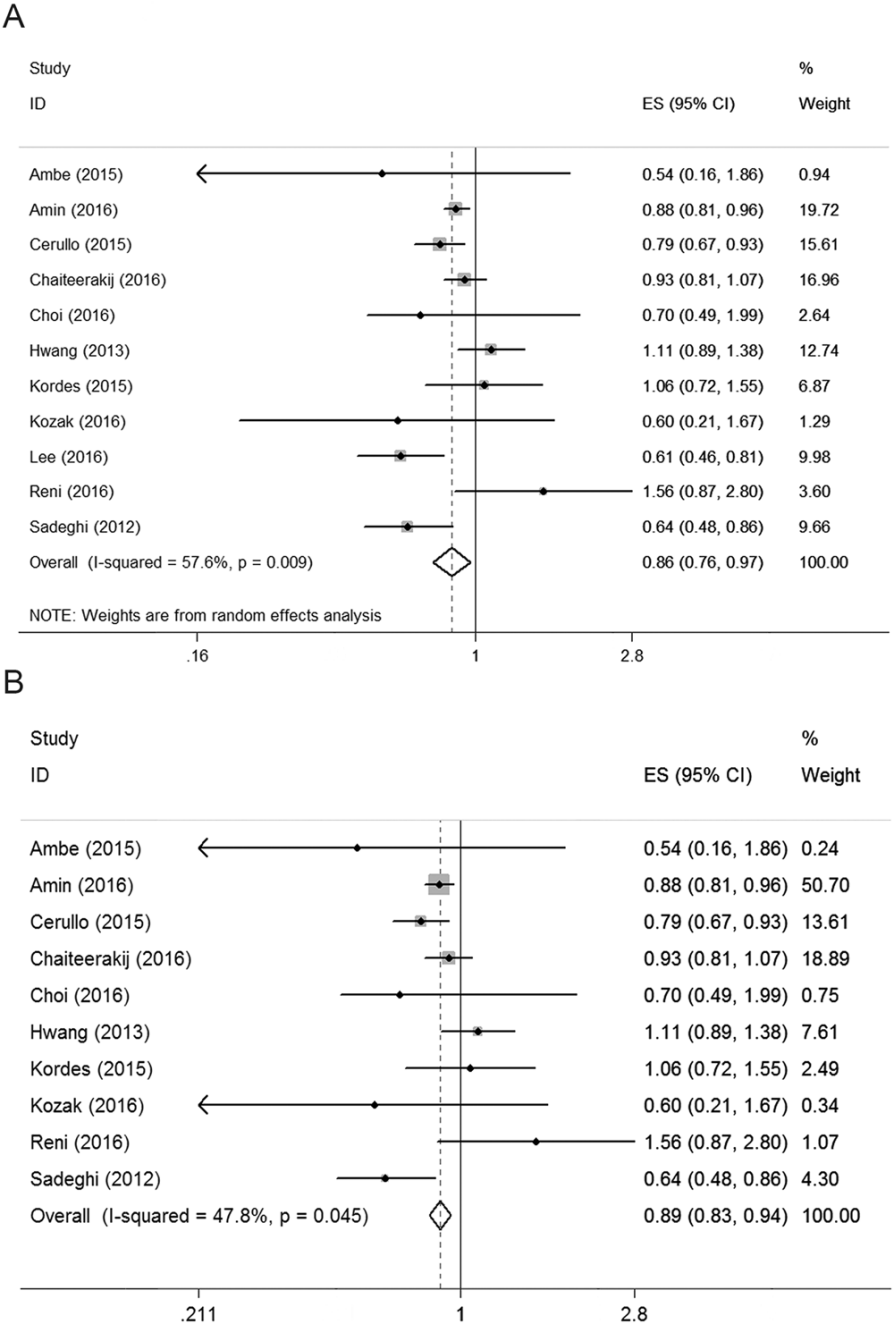
Forest plot for overall survival in all included studies (**A**) and sensitivity analysis excluded one study (**B**).

The patient survival data are shown in Fig. 2A. All nine cohort studies and two RCTs, including 2792 patients in the metformin use group, reported patient survival as an outcome. The metformin group had significantly better survival compared with the control group (HR = 0.86, 95% CI: 0.76–0.97, *P* = 0.01). In addition, there was significant heterogeneity between the studies (χ^2^ = 23.61, df = 10; *P* < 0.01; *I* ^2^ = 57.6%). Sensitivity analysis was conducted by excluding one study at a time. The study by Lee *et al*. was identified as largely contributing to heterogeneity. After excluding that particular study, the inter-study heterogeneity weakened (χ^2^ = 17.23, df = 9; *P* < 0.05; *I* ^2^ = 47.8%), and the overall result was not affected (pooled HR = 0.89, 95% CI 0.83–0.94; *P* < 0.01, Fig. 2B).

### Subgroup analysis by tumor stage

**Figure 3:**
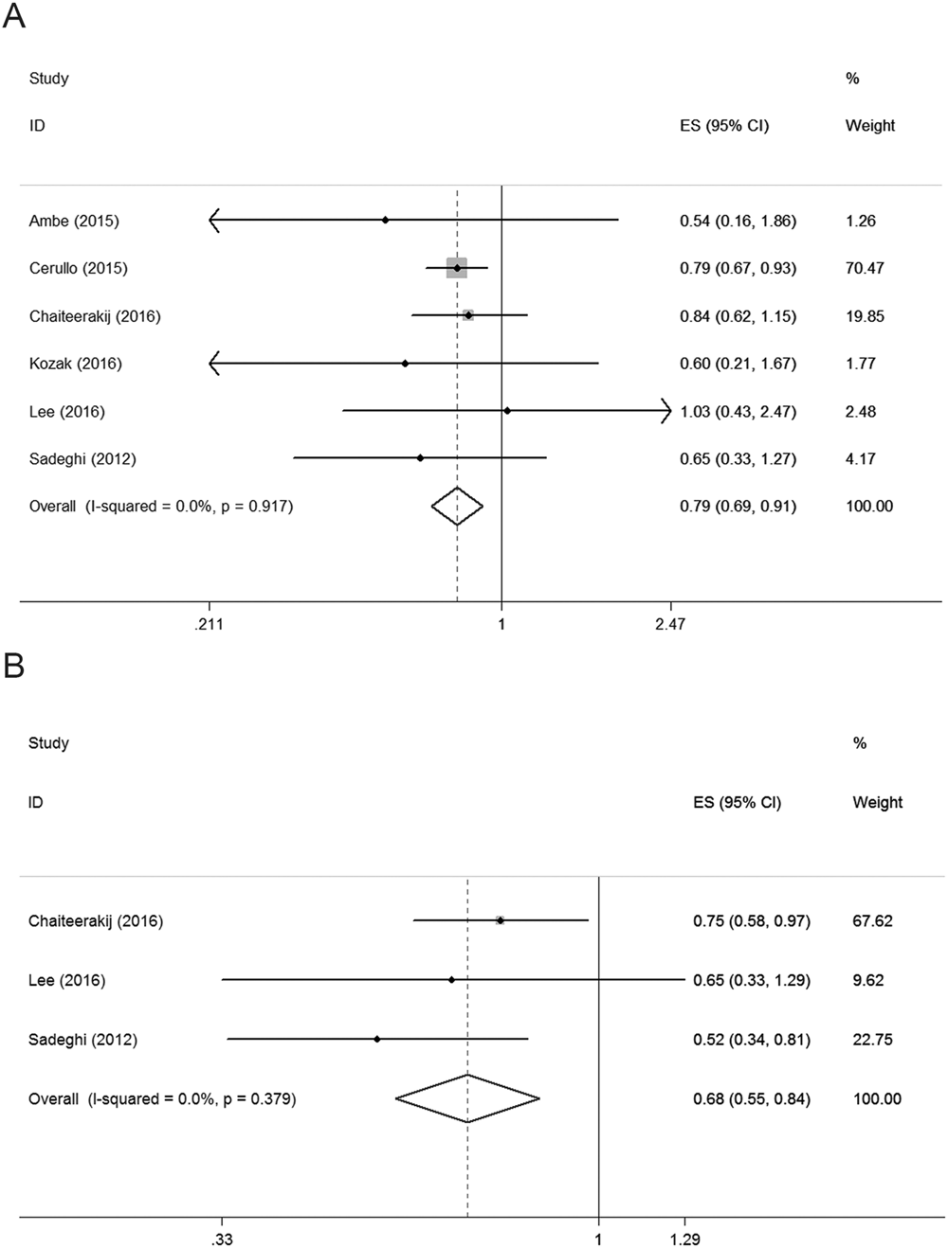
Forest plot for survival in patients at resectable stage (**A**) and locally advanced stage (**B**).

**Figure 4:**
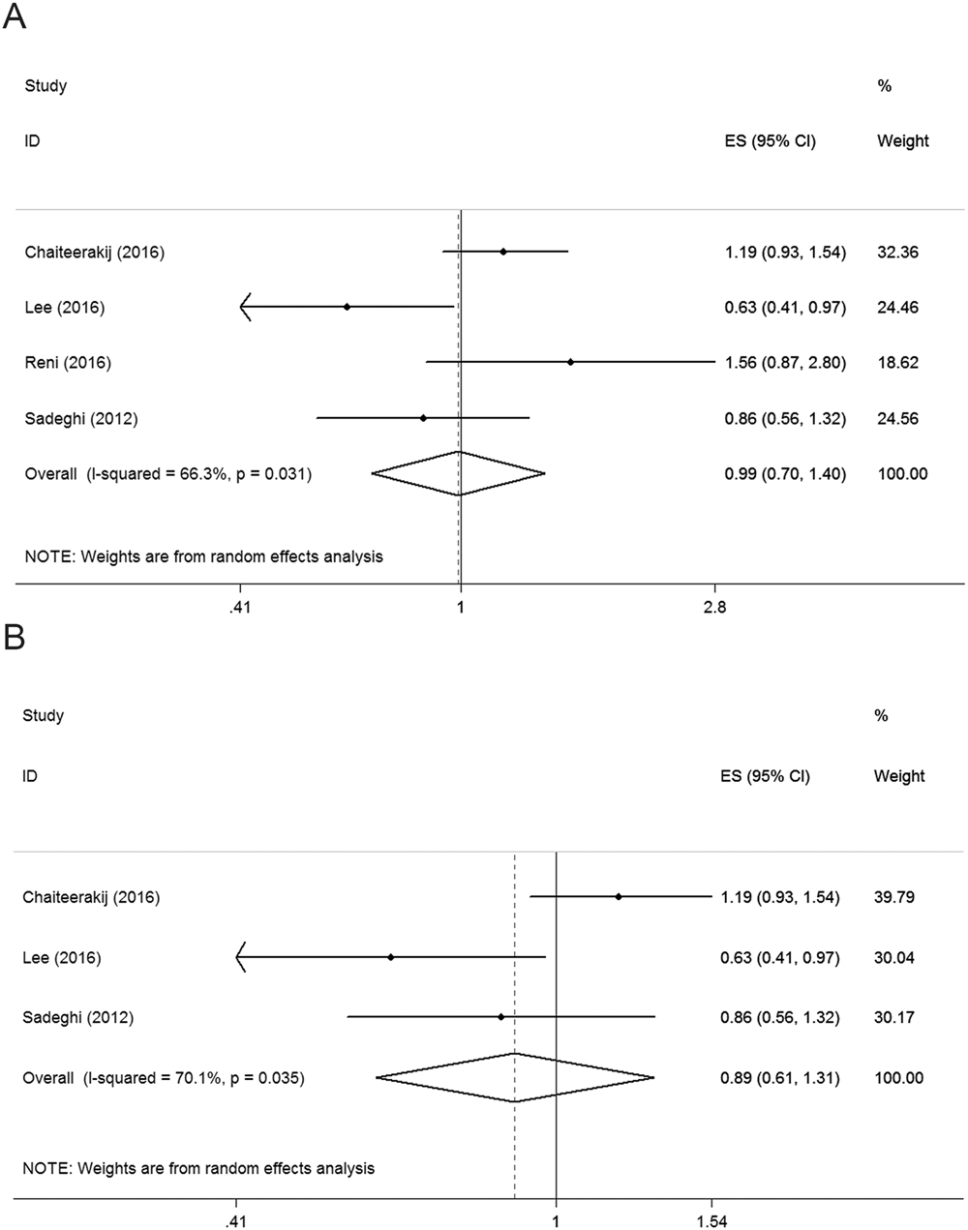
Forest plot for survival in patients at metastatic stage (**A**) with RCT, (**B**) without RCT).

Analysis of subgroups by tumor stage was performed. In the subgroup of patients at a resectable stage that included six retrospective cohort studies (4012 patients), a significantly better survival was observed in the metformin group compared to the non-metformin group (HR = 0.79, 95% CI: 0.69–0.91, *P* < 0.01, Fig. 3A). In the subgroup of patients with local advanced tumors, which included three retrospective cohort studies (548 patients), a significantly better survival was also observed in the metformin group compared to the control group (HR = 0.68, 95% CI: 0.55–0.84, *P* < 0.01, Fig. 3B). In the subgroup of metastasis patients, which included three retrospective cohort studies and one RCT (319 patients), the difference between the metformin and control group was not significant when the RCT was included (HR = 0.99, 95% CI: 0.70–1.40, *P* = 0.95, Fig. 4A) or not (HR = 0.89, 95% CI: 0.61–1.31, *P* = 0.56, Fig. 4B).

### Publication bias

No significant publication bias was found by funnel plots and Begg’s test (*P* = 0.64, Supplementary Figure S2).

## Discussion

In this meta-analysis of cohort studies and RCTs investigating metformin in patients with pancreatic cancer, we demonstrated that metformin significantly benefited patients with pancreatic cancer with regard to survival. However, the later the tumor stage, the more obscure the effect of metformin with respect to its efficacy.

The antitumor effect of metformin has been suggested both by epidemiological data and laboratory studies, which provided the rationale for the clinical use of metformin in cancer treatment. Zhang *et al*. reported a meta-analysis of four cohort studies with significant heterogeneity, and the source of heterogeneity was not further analyzed. In recent years, a number of cohort studies and two RCTs have been conducted on the same subject, and the RCTs showed a different trend with respect to the effect of metformin on pancreatic cancer patients compared with the cohort studies. Therefore, we conducted this new meta-analysis on all nine cohort studies and two RCTs with particular focus on the source of the heterogeneity. The results indicate that tumor stage may contribute greatly to the heterogeneity of the results.

Although the results of the two RCTs were inconsistent with a meta-analysis of previous cohort studies i.e., the benefit of metformin on pancreatic cancer demonstrated in the meta-analysis was not corroborated in the RCTs, this can be explained by the heterogeneity for assigned patients. As shown in Figs 3 and 4, the meta-analysis showed that metformin only benefits patients without metastatic disease depending on whether the RCTs were included, but the assigned patients in both of the RCTs included metastatic pancreatic cancer. The trial of Reni *et al*. included only metastatic pancreatic cancer patients and the trial of Kordes *et al*. included both locally advanced and metastatic pancreatic cancer patients. We tentatively postulate that the involvement of metastatic pancreatic cancer patients contributed greatly to the results. Due to the significant heterogeneity between subgroups and the lack of RCTs with resectable pancreatic cancer patients included, we also retrieved clinical trials focusing on the effect of metformin on other solid tumors from PubMed to serve as a reference. Four trials showed that metformin benefited patients with resectable cancer in colorectal cancer^35^, gastric cancer^36^, endometrial adenocarcinoma^37^, and bladder cancer^38^, while one RCT showed no beneficial evidence for metformin on metastatic cancer in non-small cell lung cancer^39^. These findings also suggest that tumor stage plays an important role in the heterogeneity between studies.

The exact reason for the heterogeneity between different stages of tumors is unknown. We tentatively speculate that the concentration of metformin in the tumor tissue may play an important role. In patients with metastatic disease, tumor *per se* was always dense irrespective of large tumor burden, thus the concentration of metformin in neoplastic tissue meant that it was difficult to accumulate drug at a therapeutic level. Therefore, the treatment effect on patients in this group is difficult to explain. However, in patients with resection, plasma metformin might act directly on circulating cancer cells and micro-lesions, which are not macroscopic. Hence, patients in this group had an improved survival with metformin. Furthermore, Kordes *et al*. and Cerullo *et al*. also observed an improved survival in patients treated with metformin in a dose-dependent manner, which means that survival benefit increased with a rising daily dose. The improved survival of patients with a higher daily dose may indirectly reflect the influence of insufficient concentration of metformin in neoplastic tissue and its subsequent antitumor effect.

The two RCTs, which are more robust than cohort studies in terms of methodology, showed no beneficial effect of metformin. Due to these disappointing results, which were inconsistent with cohort studies and laboratory studies, the impact of metformin on the survival of pancreatic cancer patients seems very limited. However, the tumor stage varied in clinical trials. Moreover, results of cohort studies were consistent with RCTs, if only metastasis cancer patients were involved. Therefore, the results of the two RCTs are not sufficient to infer that metformin has no beneficial effect on the treatment of patients with pancreatic cancer, especially resectable cancer. Furthermore, perhaps what is more important in the trial by Reni and colleagues is that they evaluated not only the effect of metformin on survival but also the drug’s toxicity to patients. Their study demonstrated that metformin combined with chemotherapeutic agents was well tolerated, which has paved the way for subsequent studies.

The use of metformin in pancreatic cancer is similar to that of immunomodulatory treatment of critical illnesses. Both treatments have been documented to be beneficial in animal experiments and primary clinical studies but subsequent randomized clinical trials have demonstrated no beneficial effect or adverse effects, which made the treatments questionable^40^. However, subsequent studies using immunomodulators have indicated that certain populations of patients do benefit from these treatments^41, 42^. Similarly, since the trials were heterogeneous with regards to the tumor stage of pancreatic cancer, it is still possible that patients with resectable or locally advanced pancreatic cancer can benefit from metformin. According to the present meta-analysis and systematic review, metformin has the potential to benefit pancreatic cancer patients, especially those patients with tumor at an early stage. Therefore, further clinical trials should focus on patients with resectable cancer.

In interpreting the results of this meta-analysis, both the limited number of available studies and the methodological quality of the included trials should be noted. Of the 11 studies included, only two studies were RCTs, and the methodological quality of one trial was relatively low. Considerable heterogeneity was observed among these trials with regards to the effect of metformin. However, the limited number of studies hampered the power of the subgroup analysis conducted due to heterogeneity. Therefore, there is clearly a need for further investigations that include robust methods and analysis.

## Materials and Methods

### Systematic literature search

A systematic electronic search was conducted in the PubMed, Embase, Cochrane, and Web of Science databases. The searches were restricted to human disease published before July 23, 2016. The keywords “metformin” and “pancreatic cancer” were used but language restriction was not imposed. Search results were imported into a database and the duplicate records were removed. The titles and abstracts were then screened for relevance. If more than one article was published by the same research team on the same topic, only the most recent article was selected in our study. Next, the full-text papers were checked for eligibility. If the paper was not published in English, we sent an e-mail to the author to ask for an English edition. If an English edition was provided, the study was included. If not, it was eliminated. There was no need for participant consent in this review, since studies included were published without personal identifiable information.

### Inclusion and exclusion criteria

We included all human cohort studies and RCTs that investigated the effects of metformin on survival of pancreatic cancer patients. We excluded: (1) cohorts that had no control group, (2) studies that did not report the essential outcomes (survival with HR and 95% CI), and (3) cohorts that included patients with other types of pancreatic tumor (e.g., pancreatic neuroendocrine tumors) and those that did not report the results for pancreatic cancer (adenocarcinoma) separately. The literature retrieval and screening were conducted respectively by two authors (LXG and LT), and a third author (GSM) was consulted when there was uncertainty or disagreement.

### Data extraction

From the included studies, the following information was extracted: design of study, tumor stage, sample size, treatment strategy or exposure to metformin in study group, intervention in the control group, and survival. If the original data for the meta-analysis were not provided directly in the text, then we sent an e-mail to the corresponding author or extracted data from Kaplan-Meier curves in a previously described way^43, 44^.

### Quality assessment

The internal validity for RCTs was determined using the Jadad scale and risk bias tool of the Cochrane Collaboration. The Newcastle-Ottawa Scale (NOS), including eight quality criteria, was applied to evaluate the quality of retrospective cohort studies.

### Statistical analysis

All summarized data were analyzed using Stata, Windows version 12.0. The HR with 95% CI was used as a surrogate for survival effect. Heterogeneity between different trials was assessed by the chi-square test, and the extent of inconsistency was evaluated by the *I* ^2^ statistic. A Mantel-Haenszel random-effects model was used for data if heterogeneity was significant; otherwise, the fixed-effects model was applied. Sensitivity analysis was conducted to assess the impact of each study on the pooled HR and heterogeneity by removing one study at a time. Publication bias was investigated by funnel plot analysis using Begg’s regression test if sufficient studies were included. Two-tailed *P*-values < 0.05 were considered statistically significant.

### Data availability

All data generated or analyzed during this study are included in this published article (and its Supplementary Information files).

## Electronic supplementary material

supplementary tables and figures
