# Supplementary material for: The effect of metformin on survival of patients with pancreatic cancer: a meta-analysis
Source: Sci Rep. 2017 Jul 19;7:5825. doi: 10.1038/s41598-017-06207-x (PMC5517652; doi:10.1038/s41598-017-06207-x)

The effect of metformin on survival of patients with pancreatic cancer: a meta-analysis

Xiaogang Li[1,3], Tong Li[2], Zhiqiang Liu[1], Shanmiao Gou[1*], Chunyou Wang[1*]

Supplementary Table S1 Quality assessment of the two RCTs.

Study	Blinding method	Randomization	Generation of random sequence	Withdrawals	Allocation concealment	Jadad score
Kordes 2016	Double-blinded	Yes	Computer-generated permuted-block randomisation	0/0	Yes	5
Reni 2015	None	Yes	Unclear	0/0	Unclear	2

Supplementary Table S2 NOS scores of the nine retrospective cohort studies.

Study	Selection				Comparability	Outcome			Overall
	Representative of cases	*Selection of controls*	*Ascertainment of exposure*	*Outcomes present at start of study*	*Comparability the design or analysis*	*Assessment of outcome*	*Adequate follow-up time（≧1 year）*	*Adequacy of follow up (≧ 80%)*	**Quality**
Ambe 2015	1	1	1	–	2	1	1	–	7
Amin 2016	1	1	1	–	2	1	1	–	7
Cerullo 2016	1	1	1	1	2	1	1	–	8
Chaiteerakij 2016	1	1	1	1	2	1	1	–	8
Choi 2016	1	1	1	–	2	1	1	–	7
Hwang 2013	1	1	1	–	2	1	1	–	7
Kozak 2016	1	1	1	1	2	1	1	–	8
Lee 2016	1	1	1	–	2	1	1	–	7
Sadeghi 2012	1	1	1	1	2	1	1	–	8

Supplementary Figure S1. Bias assessment for the two RCTs.

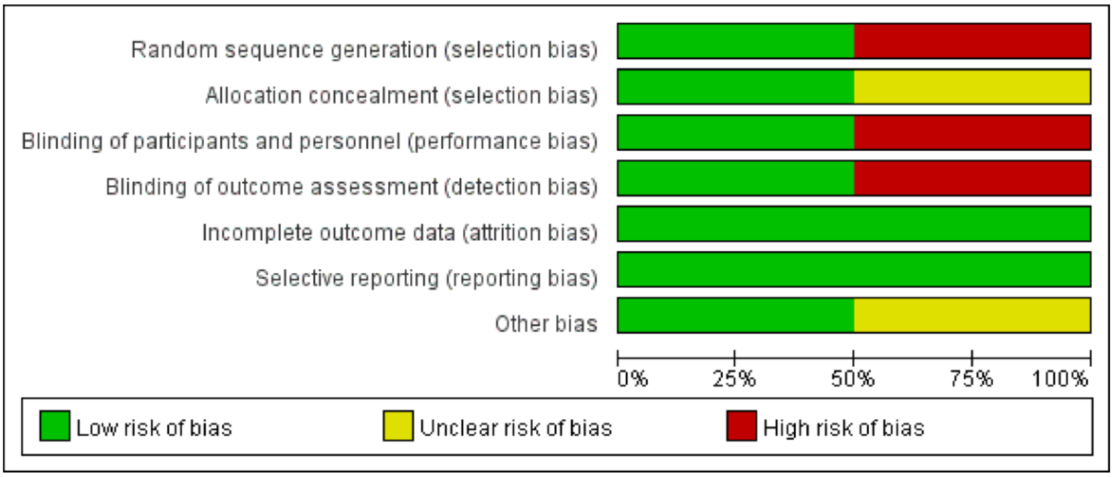

Supplementary Figure S2. Funnel plot with Begg's test for publication bias.

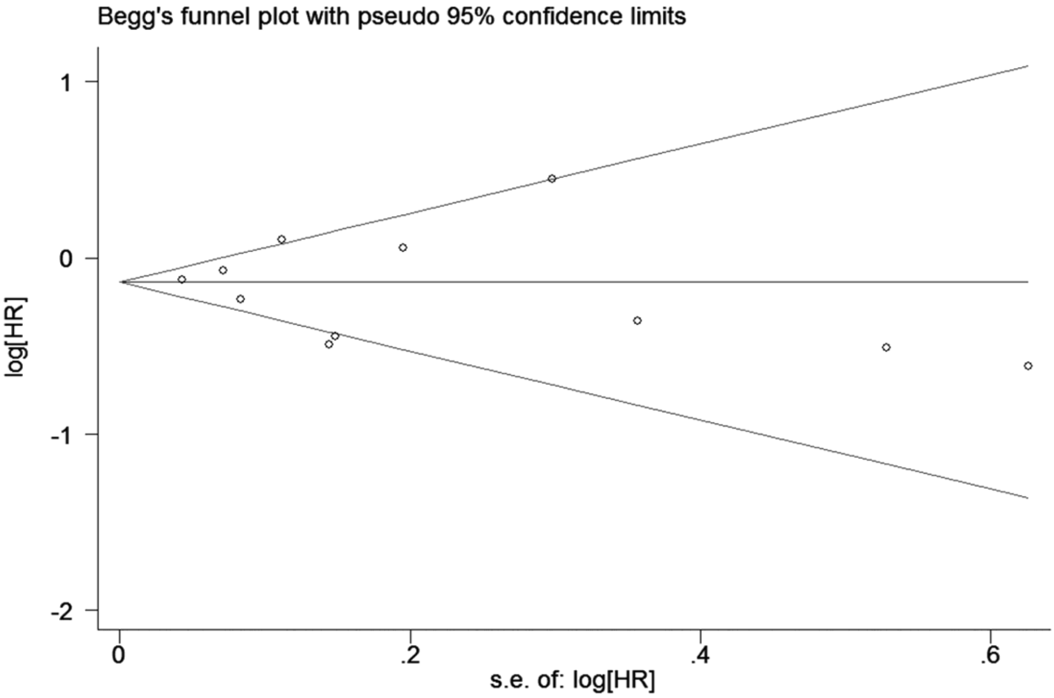

Supplement: Supplementary file 1 — supplementary tables and figures [file 41598_2017_6207_MOESM1_ESM.pdf]
